# Nanomedicine: Advancement in healthcare

**DOI:** 10.1016/j.amsu.2022.104078

**Published:** 2022-06-24

**Authors:** Arpit Mago, Muhammad Junaid Tahir, Muhammad Arslan Khan, Khabab Abbasher Hussien Mohamed Ahmed, Muhammad Usman Munir

**Affiliations:** Jawaharlal Nehru Medical College, Belgaum Karnataka, India; Lahore General Hospital, Lahore, 54000, Pakistan; Department of Pharmaceutical Sciences, University of Lahore Teaching Hospital, Lahore, Pakistan; Faculty of Medicine, University of Khartoum, Khartoum, Sudan; Department of Pharmaceutical Chemistry, College of Pharmacy, Jouf University, Sakaka, Aljouf, 72388, Saudi Arabia

**Keywords:** Nanomedicine, Antimicrobial resistance, Drug delivery, Therapeutic effect

## Abstract

With recent research and clinical advancements progressing, great strides have been made in treating both infectious and non-infectious diseases more specifically and with limited side effects. Nanotechnology in medicine has revolutionized drug delivery and improved treatment options. While they have increased efficacy, bioavailability, dose–response, targeting ability, combat antimicrobial resistance, and enhanced safety, the field is often unexplored and limited to academic institutional interests. Government support, specific flagship programmes, and more significant investments in this field could yield promising results with a greater understanding of its usage and related adverse effects.

Nanotechnology involves the study of matter at an atomic and molecular level. Its application in medicine, referred to as nanomedicine, has gained wide attention recently due to its diagnostic and therapeutic use. Nanomedicine includes the development of nanoparticles, nanostructured surfaces, and nano analytic probes for the treatment of diseases [1]. Several researches have been performed to explore more in this vast and emerging field. Nanomedicine via nanoparticles offers exciting opportunities to achieve innovation in health care. Nanoparticles, i.e., nanoscale sized particles possess characteristic features: optical, chemical, magnetic, electrical, and biological properties [2].

Antimicrobial resistance (AMR) poses a threat to the treatment and prevention of microbial infections, and measures to control its emergence need immediate attention. Factors such as inadequate and inappropriate antibiotic use have rapidly spread AMR in humankind [3]. On the other hand, nanomedicine has revolutionized medicine as nanoparticles treat even multidrug-resistant bacteria. Nanoparticles owing to their nano size, different shapes, roughness, zeta potential, possess efficient antibacterial action through their antibacterial mechanism need to be researched more in detail [4].

Conventional drug delivery mechanisms have been associated with lot of shortcomings from issues with stability of the drugs, poor solubility, reduced absorption, bioavailability, higher side effects as well as non targeted drug delivery that warrants development of newer drug delivery mechanisms using nanoparticles [7].

Nanomedicine has gained prominence significantly recently as their structural morphology ensures targeted delivery and controlled release [5]. Besides, the very small particle size also facilitates its entry into the target cells ensuring optimal action [6]. The advantages include the high drug loading capacity of nanoparticles and their tendency to reach the desired site without affecting neighbouring healthy tissue. More work has to be initiated to improve their efficiency, permeability and retention in the target sites besides development of mechanisms that can help in regulating the proportion of different nanoparticles released in multidrug combinations [8].

In today's era, extensive research and growth is seen in nanomedicine and India is also contributing significantly in this field, synergising academia, research institutions, and industry. Government organizations like the Defence Research and Development Organization (DRDO), the Department of Biotechnology (DBT), as well our country's premier research Institution Indian Council of Medical Research (ICMR) have taken lead in fostering research along and innovation in nanomedicine with active support and involvement of the best of scientific minds at private universities and corporations.

Yet, the commercial availability is limited by a lack of regulatory framework, policies and standards by the government, with significant research limited in academic institutions. In depth searcges into the different databases revealed about 19 different health-care products employing the use of nanotechnology in clinical trial phase. But significant strides have already been made by introduction of nanomedicine based products in the Indian Healthcare sector including commonly used pain relieving, anti inflammatory gels like Volini that contain diclofenac nanoparticles.Besides this paclitaxel containing drugs in bound form with alvumin have also been involved in the treatment of breast cancer [9].

Amongst other potential applications of nanotechnology in medicine are nano-adjuvants, nano-knife, nanoshells, and carbon nanotubes [10] as all these have broadened the field of nanomedicine both in therapeutics and diagnostics.

While initial results from research and trials show promising results from nanomedicine related products, its clinical safety and large scale use would require more in depth research and analysis to make it a commercial success in the health sector.

## Financial Support

No financial support was acquired for this article.

## Author contributions

A.M conceived the idea, A.M, M.J.T, and M.U.M wrote up a letter and finally M.U.M, M.A.K, K.A.H and M.J.T reviewed and provided input. All authors approved the final version of the manuscript.

## Declaration of competing interest

The authors declare no conflict of interest.
